# Characterization of differential properties of rabbit tendon stem cells and tenocytes

**DOI:** 10.1186/1471-2474-11-10

**Published:** 2010-01-18

**Authors:** Jianying Zhang, James H-C Wang

**Affiliations:** 1 MechanoBiology Laboratory, Departments of Orthopaedic Surgery, Bioengineering, and Mechanical Engineering and Materials Science, University of Pittsburgh, Pittsburgh, PA 15213, USA

## Abstract

**Background:**

Tendons are traditionally thought to consist of tenocytes only, the resident cells of tendons; however, a recent study has demonstrated that human and mouse tendons also contain stem cells, referred to as tendon stem/progenitor cells (TSCs). However, the differential properties of TSCs and tenocytes remain largely undefined. This study aims to characterize the properties of these tendon cells derived from rabbits.

**Methods:**

TSCs and tenocytes were isolated from patellar and Achilles tendons of rabbits. The differentiation potential and cell marker expression of the two types of cells were examined using histochemical, immunohistochemical, and qRT-PCR analysis as well as *in vivo *implantation. In addition, morphology, colony formation, and proliferation of TSCs and tenocytes were also compared.

**Results:**

It was found that TSCs were able to differentiate into adipocytes, chondrocytes, and osteocytes *in vitro*, and form tendon-like, cartilage-like, and bone-like tissues *in vivo*. In contrast, tenocytes had little such differentiation potential. Moreover, TSCs expressed the stem cell markers Oct-4, SSEA-4, and nucleostemin, whereas tenocytes expressed none of these markers. Morphologically, TSCs possessed smaller cell bodies and larger nuclei than ordinary tenocytes and had cobblestone-like morphology in confluent culture whereas tenocytes were highly elongated. TSCs also proliferated more quickly than tenocytes in culture. Additionally, TSCs from patellar tendons formed more numerous and larger colonies and proliferated more rapidly than TSCs from Achilles tendons.

**Conclusions:**

TSCs exhibit distinct properties compared to tenocytes, including differences in cell marker expression, proliferative and differentiation potential, and cell morphology in culture. Future research should investigate the mechanobiology of TSCs and explore the possibility of using TSCs to more effectively repair or regenerate injured tendons.

## Background

The function of tendons is to transmit muscular forces to bone, permitting joint motion and subsequent body movement. Therefore, tendons are constantly subjected to large mechanical loads and, as a result, are prone to acute injuries. For example, during sports activities, acute partial tendon injuries are common [1]. Injured tendons heal slowly and often result in the formation of inferior scar tissue or fibrous adhesions, which increases the risk of re-injury at the repair site. Tendons are also susceptible to loading-induced tendinopathy, a broad term describing tendon inflammation and degenerative changes [2]. Despite its high prevalence, the pathogenic mechanisms of tendinopathy are unclear and consequently, current treatments are largely palliative. In fact, the restoration of normal structure and function of injured tendons represents one of the most challenging areas in orthopaedic medicine.

In recent years, a tissue engineering approach has been sought to improve the structure and function of injured tendons using stem cell therapy [3]. A common source of stem cells used in tissue engineered repair of injured tissues is bone marrow mesenchymal stem cells (BMSCs). BMSCs are multipotent cells that can differentiate into several cell types [4], including chondrocytes and osteoblasts. BMSC therapy therefore offers a promising treatment option for damaged cartilage and bone [5]. BMSCs have also been used in the repair of injured tendons, but in many cases ectopic bone was formed within tendons in a rabbit tendon injury model [6]. Besides BMSCs, adult cells, such as dermal fibroblasts and autologous tenocytes, have also been used to treat injured tendons, meeting with varying degrees of success [7,8]. Therefore, the development of new effective cell therapies for the restoration of normal tendon structure and function is highly desirable, but progress has been hindered by a lack of characterization of tendon cells. Recently, remarkable progress has been made with the identification of human and mouse tendon stem/progenitor cells (TSCs) [9]. TSCs are characterized by their multidifferentiation potential, including differentiation into adipocytes, chondrocytes, and osteocytes. However, de Mos et al. showed that tendon-derived fibroblasts (TDFs) from adolescent non-degenerative human hamstring tendons are able to differentiate into adipocytes, chondrocytes, and osteocytes [10], suggesting that tendon fibroblasts or tenocytes may have trans-differentiation potential. As tendons contain predominantly tenocytes, in addition to newly identified TSCs, these previous studies raise the question of whether TSCs and tenocytes share common properties in their phenotypes or whether they are completely different types of cells with different characteristics. We hypothesized that TSCs differ from tenocytes in differentiation potential, cell marker expression, morphology, and proliferative potential. To test this hypothesis, we used young rabbits to isolate TSCs and tenocytes from patellar and Achilles tendons for characterizing their cellular properties. No studies to date have reported TSCs in rabbits, which are often used as an animal model for the study of tendon healing and biomechanics due to their relatively large size and low cost for *in vivo *experiments [11,12].

## Methods

### Isolation of TSCs and tenocytes

The cell isolation method was based on a previous study [9]. Fifteen female New Zealand white rabbits (8-10 week-old, 3.0 - 4.0 kg) were used in all experiments. The protocol for use of the rabbits was approved by the IACUC of the University of Pittsburgh. All rabbits were fully sedated using intra-muscular Ketamine (10 mg/kg) and Xylazine (3 mg/kg) injection and were then sacrificed. After sacrifice, rabbit patellar and Achilles tendons were dissected. The middle portions of tendons, which were utilized for cell culture, were obtained by cutting the tendon samples 5 mm from the tendon-bone insertion and tendon-muscle junction. The tendon sheath and surrounding paratenon were removed, and the middle tendon portion tissues were then weighed and minced into small pieces (1 mm × 1 mm × 1 mm). Each 100 mg tissue sample was digested with 3 mg collagenase type I (Worthington Biochemical Corporation, Lakewood, NJ) and 4 mg dispase (StemCell technologies Inc., Vancouver, BC, Canada) in 1 ml phosphate-buffered saline (PBS) at 37°C for 1 hr. The suspensions were centrifuged at 1,500 g for 15 min, and the supernatant was discarded. The remaining cell pellet was re-suspended in growth medium consisting of Dulbecco's modified Eagle's medium (DMEM; Lonza, Walkersville, MD) supplemented with 20% fetal bovine serum (FBS; Atlanta Biologicals, Lawrenceville, GA), 100 μM 2-mercaptoethanol (Sigma-Aldrich, http://www.sigmaaldrich.com), 100 U/ml penicillin and 100 μg/ml streptomycin (Atlanta Biologicals, Lawrenceville, GA). A single-cell suspension was obtained by diluting the suspension to 1 cell/μl and then cultured in either a 96 well plate or T25 flasks at 37°C with 5% CO_2_. After 8-10 days in culture, patellar TSCs (PTSCs) and Achilles TSCs (ATSCs) formed colonies on the culture surface of the plate or flask. The cell colonies were stained with methyl violet. Colony numbers and total cell number of all colonies were counted using a hemocytometer.

In separate cultures, individual cell colonies were detached by local application of trypsin under microscopic visualization. The detached cell colonies were collected using a micropipette and transferred to individual T25 flasks for further culture. After removal of cell colonies, tenocytes, which were spread around, remained in culture plates. These cells, which were elongated in shape, were cultured further with the addition of regular growth medium (DMEM plus 10% FBS).

TSC and tenocyte proliferation was assessed with population doubling time (PDT), defined as the total culture time divided by the number of generations. The number of generations was expressed as log_2_N_c_/N_0_, where N_0 _is the population of the cells seeded initially, and N_c _is the population at confluence.

### *In vitro *differentiation experiment

The multi-differentiation potential of the TSCs was tested *in vitro *for adipogenesis, chondrogenesis, and osteogenesis. TSCs at passage 1 were seeded in a 6-well plate at a density of 2.4 × 10^5 ^cells/well in basic growth medium (10% heat inactivated FBS, 100 U/ml penicillin and 100 μg/ml streptomycin in DMEM-low glucose). To test adipogenic potential, TSCs were cultured in adipogenic induction medium (Millipore, Cat. # SCR026) consisting of basic growth medium supplemented with 1 μM dexamethasone, 10 μg/ml insulin, 100 μM indomethacin, and 0.5 mM isobutylmethylxanthine (IBMX). As a test of chondrogenic potential, TSCs were cultured in basic growth medium plus 40 μg/ml proline, 39 ng/ml dexamethasone, 10 ng/ml TGF-β 3, 50 μg/ml ascorbate 2-phosphate, 100 μg/ml sodium pyruvate, and 50 mg/ml insulin-transferrin-selenious acid mix (ITS) from BD Bioscience (Bedford, MA). Finally, osteogenic potential was tested by culturing TSCs in osteogenic induction medium (Millipore Cat. # SCR028) consisting of basic growth medium augmented with 0.1 μM dexamethasone, 0.2 mM ascorbic 2-phosphate, and 10 mM glycerol 2-phosphate. TSCs cultured in basic growth medium were used for control cells. To assay adipogenesis, chondrogenesis, and osteogenesis of TSCs, Oil Red O, Safranin O, and Alizarin Red S assays (see below), respectively, were used.

In addition to assessing differentiation potential of TSCs, the possible trans-differentiation potential of patellar tenocytes (PTs) and Achilles tenocytes (ATs) was also tested by the same assays used for testing TSCs. PTs and ATs were grown in the same culture conditions as TSCs.

#### Oil red O assay

After culturing in adipogenic medium for 21 days, differentiated adipocytes were detected by an Oil Red O assay. In short, the medium was removed from the cell culture plates, and the cells were washed with PBS 3 times each for 5 min. The cells were then fixed using 4% paraformaldehyde for 40 min at room temperature. Subsequently, the cells were washed with PBS 3 times each for 5 min, then water 2 times each for 5 min, and finally incubated with a 0.36% Oil Red O solution (Millipore, Cat. # 90358) for 50 min, followed by washing 3 times with water. Stained samples were examined on an inverted microscope (Nikon eclipse, TE2000-U); images were obtained by a CCD (charge-coupled device) camera on the microscope and analyzed by SPOT™ imaging software (Diagnostic Instruments, Inc., Sterling Heights, MI). Stained lipid droplets of the adipocytes appeared red.

#### Safranin O assay

Chondrogenesis was evaluated by Safranin O assay. Cells cultured with chondrogenic differentiation medium for 21 days were fixed in ice cold ethanol for 1 hr, rinsed with distilled water 2 times each for 5 min, and stained at room temperature for 30 min with Safranin O solution (Sigma, St. Louis; Cat. # HT904). The cells were rinsed 5 times with distilled water. The stained cells were examined with an inverted microscope (Nikon eclipse, TE2000-U), and images were taken with a CCD camera, followed by image analysis with SPOT™ imaging software. The stained glycosaminoglycans (GAG)-rich matrix produced by chondrocytes appeared red.

#### Alizarin red S assay

The osteogenesis of TSCs was assessed by Alizarin Red S assay. Cells cultured in osteogenic differentiation medium for 21 days were fixed in chilled 70% ethanol for 1 hr, rinsed with distilled water twice each for 5 min, and stained with Alizarin Red S (Millipore, Cat. # 2003999) at room temperature for 30 min. The stained cells were examined on an inverted microscope as described above, with images being taken by a CCD camera and analyzed by SPOT™ imaging software. The stained osteocytes that contain mineral deposits appeared orange-red.

#### Quantitative real-time RT-PCR (qRT-PCR) for gene analysis

The specific gene expression of differentiated TSCs was determined using qRT-PCR. Total RNA was extracted using an RNeasy Mini Kit with an on-column DNase I digest (Qiagen, http://www.qiagen.com). First-strand cDNA was synthesized in a 20 μl reaction from 1 μg total RNA by reverse transcription with SuperScript II (Invitrogen, http://www.invitrogen.com). The conditions for the cDNA synthesis were: 65°C for 5 min and cooling 1 min at 4°C, then 42°C for 50 min, 72°C for 15 min. The qRT-PCR was carried out using QIAGEN QuantiTect SYBR Green PCR Kit (Qiagen) [13]. In a 50 μl PCR reaction mixture, 2 μl cDNA (total 100 ng RNA) were amplified in a Chromo 4 Detector (MJ Research). Rabbit-specific primers were used for collagen type I, collagen type II, peroxisome proliferators-activated receptor γ (PPARγ), Sox9, and Runx2. Glyceraldehyde-3-phosphate dehydrogenase (GAPDH) was used as an internal control. The forward and reverse primer sequences and the resultant products were designed according to published methods [14,15] and are listed in Table 1. All primers were synthesized by Invitrogen. After an initial denaturation for 10 min at 95°C, PCR was performed for 30 cycles for GAPDH, collagen type I and II, and 40 cycles for PPARγ. Each cycle consisted of denaturation for 50 seconds at 95°C, followed by annealing for 50 seconds at 58°C for GAPDH, and collagen I and II, but at 56°C for Sox9, Runx2, and PPARγ. At least three independent experiments were performed to obtain relative expression levels of each gene.

**Table 1:** Primers for qRT-PCT analysis

Gene	Size (bp)	Primers	Type	Tm	Gene Bank #	Ref.
Collagen I	81	5'-CTG ACT GGA AGA GCG GAG AGT AC-3'	Forward	63°C	AY633663	[14]
		5'-CCA TGT CGC AGA AGA CCT TGA-3'	Reverse			
Collagen II	84	5'-TGG GTG TTC TAT TTA TTT ATT GTC TTC CT-3'	Forward	63°C	S83370	[14]
		5'-GCG TTG GAC TCA CAC CAG TTA GT-3'	Reverse			
Sox9	79	5'-AGT ACC CGC ACC TGC ACA AC-3'	Forward	59°C	AY598935	[14]
		5'-CGC TTC TCG CTC TCG TTC AG-3'	Reverse			
Runx2	70	5'-TGA TGA CAC TGC CAC CTC TGA-3'	Forward	58°C	AY598934	[14]
		5'-GCA CCT GCC TGG CTC TTC T-3'	Reverse			
GAPDH	107	5'-ACT TTG TGA AGC TCA TTT CCT GGT A-3'	Forward	63°C	L23961	[14]
		5'-GTG GTT TGA GGG CTC TTA CTC CTT-3'	Reverse			
PPARγ	200	5'-TGG GGA TGT CTC ATA ATG CCA-3'	Forward	59°C	AF013266	[15]
		5'-TTC CTG TCA AGA TCG CCC TCG-3'	Reverse			

### *In vivo *differentiation experiment

TSCs (5 × 10^5^) were mixed with 150 μl Matrigel (BD Biosciences) in a 24-well tissue culture plate at 4°C. After incubation for 30 min in medium (DMEM with 10% FBS and 1% penicillin/streptomycin), the gel-cells were cultured at 37°C with 5% CO_2 _for another 30 min. Human skin fibroblasts were treated in the same way as the rabbit TSCs and used as a control. The gel-cells were injected subcutaneously in the paraspinal region bilaterally in six 10-week-old female nude rats (Charles River Laboratories, Wilmington, MA). Tissue samples were harvested at 8 weeks and placed in pre-labeled base molds filled with frozen section medium (Neg 50; Richard-Allan Scientific; Kalamazoo, MI). The base mold with tissue samples was quickly immersed in liquid nitrogen cold 2-methylbutane and allowed to solidify completely. The tissue blocks were then placed on dry ice and subsequently stored in the -80°C freezer until being sectioned for histological analysis.

### Histochemical and immunohistochemical analysis of tissue sections

The tissue block was cut into 10 μm thick sections, and they were then placed on glass slides and allowed to dry overnight at room temperature. The sections were rinsed three times with PBS, fixed with 4% paraformaldehyde for 30 min, and washed with PBS three more times. The sections were histochemically stained with H&E, Alcian blue, and Alizarin Red S (all reagents were from Sigma). For immunohistochemical staining, the sections were coated with 5% goat serum and incubated for 30 min at room temperature in a humid chamber. The serum was carefully removed by aspiration and rabbit anti-collagen type I antibody (1:200; Rockland, Cat. No. 600-401-103) was applied to the sections, which were then incubated at room temperature for 2 hrs. They were washed three times with PBS, reacted with Cy3-conjugated donkey anti-rabbit IgG (1:500; Rockland, Cat. No. 611-704-127) at room temperature for 1 hr, again washed three times with PBS, and reacted with Hoechst fluorochrome 33342 (1:1000; Sigma, Cat. No. H33342) at room temperature for 5 min. Finally, the sections were washed with running cold water for 5 min, followed by a distilled water rinse. The control samples received the same treatments, except that primary antibodies were replaced with PBS.

### Immunohistochemical analysis of cell markers

Using immunocytochemistry, we examined the following stem cell markers: octamer-binding transcription factor 4 (Oct-4), stage-specific embryonic antigen-4 (SSEA-4), and nucleostemin. The TSCs were fixed with 4% paraformaldehyde in phosphate-buffered saline for 30 min at room temperature, blocked with 10% mouse serum for 1 hr at room temperature, and reacted with mouse anti-human Oct-4 (1:250; Millipore, Cat. No. MAB4401) or SSEA-4 antibody (1:500; ZYMED Laboratories, Invitrogen Immunodetection, Cat. No. 41-4000) for 1 hr at room temperature. After washing the cells with PBS, Cy3-conjugated goat anti-mouse secondary antibody (1:1000; Invitrogen Molecular Probes, Cat. No. A10521) was applied for 30 min at room temperature for Oct-4 or FITC-conjugated goat anti-mouse IgG (1:1000; BD Pharmingen, Cat. No. 554001) was applied for 30 min at room temperature for SSEA-4. A similar protocol was adopted to perform immuno-staining of nucleostemin on rabbit TSCs. The staining protocol used goat anti-human nucleostemin antibody (1:300; Neuromics, Cat. No. GT15050) and Cy3-conjugated donkey anti-goat IgG secondary antibody (1:1000; Millipore, Cat. No. AP180C). The cells were also counterstained with H33342 staining (Sigma). The stained cells were examined using fluorescence microscopy. Human skin fibroblasts (ATCC, #CRL-2703) were used as a negative control, while human patellar tendon stem cells, isolated according to the protocol by a previous study [9], were used as a positive control; both cell types received the same treatments as rabbit TSCs.

### Statistical Analysis

Data are presented as mean ± SD. At least three replicates for each experimental condition were performed, and the presented results were representative of these replicates. One-way analysis of variance (ANOVA), followed by Fisher's predicted least-square difference (PLSD) for multiple comparisons, or two tailed student *t*-test wherever applicable, was used for statistical analysis. Differences between two groups were considered significant when the p-value was less than 0.05.

## Results

### Colony formation of TSCs

**Figure 1:**
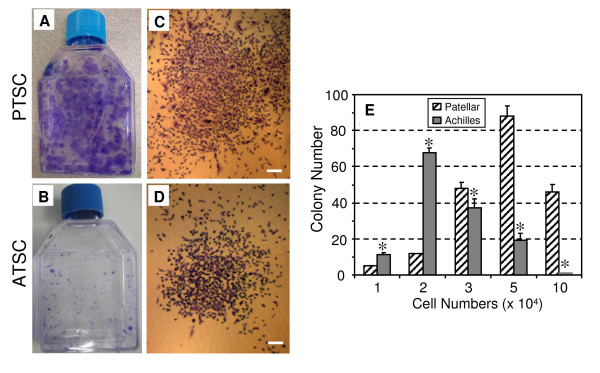
**The colony formation of rabbit tendon stem cells (TSCs)**. **A, B**. Total PTSC and ATSC colonies stained with Methyl violet at 10 days. **C, D**. Expanding colonies of PTSCs and ATSCs at 10 days, respectively. It is seen that more numerous and larger cell colonies were formed by PTSCs compared to ATSCs. **E**. Quantitative analysis of colonies formed by PTSCs and ATSCs. Colony number of PTSCs was significantly different from that of ATSCs (* p < 0.05). (Bars: 200 μm).

During the initial 2 days in culture, individual PTSCs and ATSCs were present in culture plates. These cells attached to the plate and remained quiescent for 3-5 days. The first colony was noted to form from single cells at 3 days. Numerous colonies were then formed at 10 days, and it is evident that PTSCs formed more and larger colonies than ATSCs (Figure 1A, B, C, D). The size and density of these colonies, however, were heterogeneous (Figure 1A, B), indicating unequal rates of cell proliferation among colonies.

At 14 days, both PTSCs and ATSCs formed circular colonies at a frequency of 42.1 ± 8.1 colonies and 48.3 ± 7.6 colonies per 10^5 ^viable cells, respectively. Furthermore, 50.3% of PTSC colonies consisted of 50,000 cells or more, while only 23.2% of ATSC colonies had comparable populations (Figure 1E).

### Cell multi-differentiation potential

**Figure 2:**
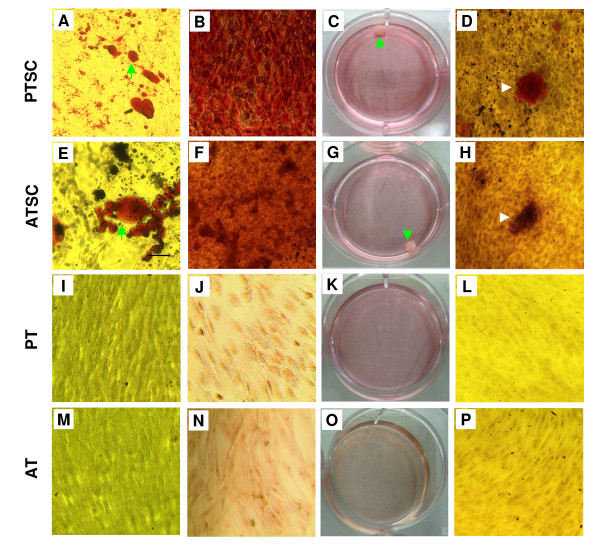
**The testing of multi-differentiation potential of TSCs and tenocytes *in vitro***. **A**. Adipogenesis of PTSCs (arrows point to lipid droplets). **B**. Chondrogenesis of PTSCs. **C**. Cartilage-like pellet (arrow) formed from PTSCs. **D**. Osteogenesis of PTSCs (An arrow points to a clustered calcium droplet). Similar multi-differentiation potential is shown for ATSCs (**E**-**H**). Patellar tenocytes (PTs) and Achilles tenocytes (ATs) were not found to exhibit such a multi-differentiation potential (**I-P**, also see text for additional descriptions of experimental results). (Magnification of microscopy: 10×).

We next examined whether TSCs were capable of differentiating into various cell lineages, a characteristic of stem cells. The potential of both PTSCs and ATSCs to undergo adipogenesis, chondrogenesis and osteogenesis was tested. When cells were cultured in adipogenic differentiation medium, cytoplasmic lipid vesicles, an indicator of adipocyte differentiation, first appeared at 7 days and the amounts of lipid production continued to increase over culture time. After 21 days, numerous lipid droplets were detected on differentiated PTSCs and ATSCs (Figure 2A, E); however, a few lipid droplets were present in the control cells, which were cultured in basic growth medium without adipogenic supplements (data not shown). Approximately 75% of PTSCs were tested positive for lipid vesicles by Oil red O staining (Figure 2A), whereas about 30% of ATSCs were positive (Figure 2E).

When cultured in chondrogenic differentiation medium, PTSCs spontaneously formed large aggregates in culture at 13 days. Single aggregates were formed from an entire monolayer, which rolled up from the edge of the culture dish and contracted to form an irregularly shaped mass. At 15 days, these initially loose aggregates became firmer in texture and more spherical in shape. This process also occurred 3 days earlier in ATSCs. Cartilage-like pellets were found in PTSCs (Figure 2C) and ATSCs (Figure 2G) that had undergone chondrogenesis. After culturing in chondrogenic medium for 21 days, PTSCs and ATSCs were stained positive for GAG-rich matrix with Safranin O assay (Figure 2B, F), whereas control cells without exposure to the chondrogenic medium were stained negative (data not shown).

Also, when PTSCs were cultured in osteogenic medium for 21 days, calcium deposits were visible and stained by Alizarin Red S assay (Figure 2D), whereas calcium deposits were rarely found in control cells without exposure to osteogenic medium (data not shown). Similarly, calcium deposits in ATSCs were found in osteogenic conditions only (Figure 2H).

We also examined the possible trans-differentiation of tenocytes. It was found that less than 5% of patellar tenocytes (PTs) and 1% of Achilles tenocytes (ATs) stained positive for lipid drops, when both PTs and ATs were cultured in adipogenesis medium and exposed to the same conditions as TSCs (Figure 2I, M). Similarly, cartilage-like pellets were not found and GAG-containing matrix was not detected by the staining of PTs or ATs (Figure 2J, K, N, O). Finally, few calcium deposits were detected after staining in PTs and ATs treated with the same conditions as TSCs (Figure 2L, P).

### Specific marker gene expression

**Figure 3:**
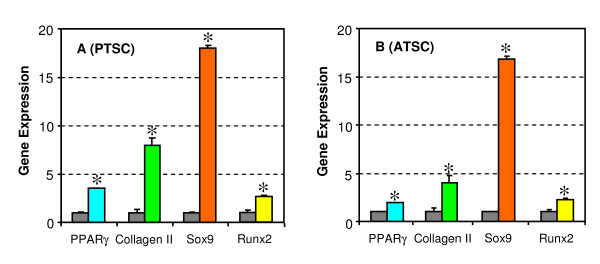
**The qRT-PCR analysis of expression of marker genes**. Rabbit PTSCs and ATSCs were differentiated into adipocytes (PPARγ), chondrocytes (collagen II and Sox9), and osteocytes (Runx2) using their respective differentiation induction media. Compared to non-differentiated, control cells (black columns) that were grown in regular growth medium, all these genes were significantly upregulated for both PTSCs (**A**) and ATSCs (**B**), albeit at different levels (* p < 0.05). Note that for real time RT-PCR analysis, the gene expression levels were normalized to GAPDH, obtained from at least three independent experiments and presented as 2^(-ΔCT)^.

To confirm that TSCs derived from rabbit patellar and Achilles tendons were differentiated into a specific lineage, qRT-PCR was used to identify specific gene markers. Adipogenesis requires the sequential action of PPARγ [16]. Indeed, PTSCs cultured in adipogenic medium for 21 days were found to exhibit significantly higher levels of PPARγ expression compared to control PTSCs (Figure 3A, columns 1 and 2). Similarly, when cultured in adipogenic medium, ATSCs also significantly up-regulated expression of PPARγ gene compared to control cells (Figure 3B, columns 1 and 2).

To confirm that TSCs were able to differentiate into chondrocytes, PTSCs and ATSCs were cultured in chondrogenic medium and found to significantly increase expression of the collagen type II gene (Figure 3A, B, columns 3 and 4). Furthermore, significantly higher levels of Sox9, a chondrogenic transcription factor, were expressed in PTSCs and ATSCs than in control cells (Figure 3A, B, columns 5 and 6).

Finally, to confirm the differentiation of TSCs into osteocytes in osteogenic medium, induction of Runx2, an osteoblast specific gene, was examined by qRT-PCR. Both PTSCs and ATSCs in osteogenic medium expressed significantly higher levels of Runx2 compared to the same cells in control medium (Figure 3A, B, columns 7 and 8).

### Multi-differentiation potential of TSCs *in vivo*

**Figure 4:**
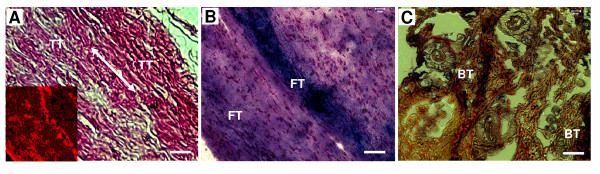
**The testing of multi-differentiation of rabbit TSCs *in vivo***. **A**. Formation of tendon-like tissue (TT) revealed by H&E staining and immunohistochemical staining for collagen type I. The collagen fibers are parallel to each other (double arrow), indicative of formation of tendon-like tissue (inset: collagen type I staining). **B**. Formation of fibrocartilage-like tissue (FT) (Alcian blue staining); and **C**. formation of bone-like tissue (BT) (Alizarin Red S staining). (Bars: 50 μm).

After TSCs were subcutaneously transplanted into nude mice, tendon-like, fibrocartilage-like, and bone-like tissues were formed at 8 weeks after imlantation (Figure 4A, B, C). However, implantation of control fibroblasts did not lead to formation of any of these tissues (data not shown).

### Morphology of TSCs and tenocytes in long term culture

**Figure 5:**
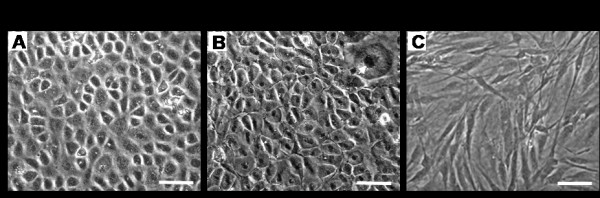
**The morphology of TSCs and tenocytes in culture**. **A, B**. PTSCs and ATSCs at passage 10 were in culture for at least 63 days, respectively. These cells were cobblestone-like in a confluent culture. **C**. Morphology of tenocytes from the same rabbit patellar tendons; similar morphology was observed in tenocytes from the Achilles tendons (not shown). These tenocytes were highly elongated in a confluent culture. (Bar: 50 μm).

Both PTSCs and ATSCs maintained a cobblestone shape after being cultured for at least 63 days and more than 10 passages (Figure 5A, B), whereas tenocytes derived from both patellar and Achilles tendons were highly elongated (Figure 5C). The marked difference in cell shape suggests that TSCs (PTSCs and ATSCs) are different type of cells from tenocytes.

### Cell marker expression

**Figure 6:**
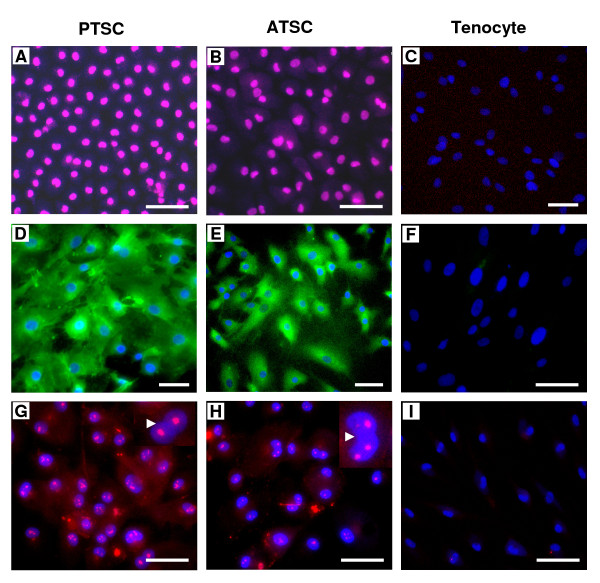
**The testing of stem cell marker expression**. **A, B**. PTSCs and ATSCs at passages 10 expressed Oct-4, respectively. **C**. No Oct-4 staining was detected on tenocytes. **D, E**. PTSCs and ATSCs expressed SSEA-4. **F**. Tenocytes were negative for SSEA-4 staining. **G, H**. PTSCs and ATSCs expressed nucleostemin. Insets show enlarged view of expressed nucleostemin in pink (arrows). **I**. Nucleostemin expression was not detected on tenocytes. (bar: 50 μm).

Immunocytochemical staining of these cells showed that both PTSCs and ATSCs in culture for more than three months at passages 10-13 expressed Oct-4 (Figure 6A, B), SSEA-4 (Figure 6D, E), and nucleostemin (Figure 6G, H). However, tenocytes exhibited an absence or very low levels of staining for these cell markers (Figure 6C, F, I), further confirming that TSCs and tenocytes are two different types of tendon cells.

### Proliferative potential of TSCs and tenocytes

**Figure 7:**
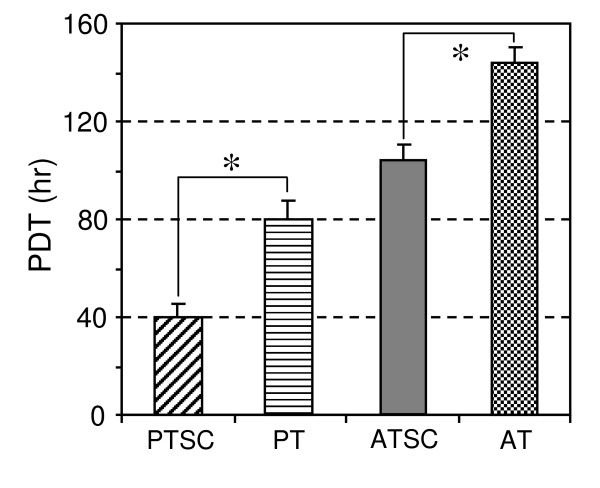
**The population doubling time (PDT) of TSCs and tenocytes**. Patellar TSCs (PTSCs), Achilles TSCs (ATSCs) at passage 2 proliferated faster than their counterparts: patellar tenocytes (PTs), and Achilles tenocytes (ATs).

The PDTs for PTSCs and PTs at passage 2 were 39.9 ± 5.5 hrs and 79.8 ± 7.3 hrs, respectively. In contrast, the PDTs for ATSCs and ATs were 103.8 ± 6.8 hrs and 143.8 ± 7.0 hrs, respectively. The results indicate that PTSCs and ATSCs proliferated faster than their counterparts (i.e. PTs and ATs), and that PTSCs proliferated faster than ATSCs (Figure 7). However, for PTSCs and ATSCs at later passages (> 12), the PDTs were increased to 187 ± 20.2 hr and 236 ± 40.1 hr, respectively, indicating that these cells were in a senescent state. Tenocytes essentially did not grow at such high passages.

## Discussion

This study aimed to determine whether TSCs and tenocytes, the two types of cells in tendons, share common properties in their phenotypes. Towards this aim, TSCs and tenocytes were isolated from rabbit patellar and Achilles tendons, and their differentiation potential, cell marker expression, morphology, and proliferative potential were examined. We found that TSCs were able to differentiate into specific lineages of cells (adipocytes, chondrocytes, and osteocytes), which was verified by detection of specific marker gene expression. The multi-differentiation potential of TSCs was further confirmed by an *in vivo *experiment, which demonstrated that implantation of TSCs *in vivo *resulted in the formation of tendon-like, fibrocartilage-like, and bone-like tissues. We also showed that TSCs expressed Oct-4, SSEA-4, and nucleostemin, which are known stem cell markers. In contrast, tenocytes from both the patellar and Achilles tendons essentially lacked trans-differentiation potential; moreover, tenocytes did not express Oct-4, SSEA-4, or nucleostemin. Morphologically, TSCs in culture differ from tenocytes in that the former exhibited a cobble-stone shape whereas the latter spread out and were highly elongated, a characteristic shape of fibroblasts in confluent conditions. Finally, TSCs proliferated significantly faster than tenocytes in culture.

The finding that TSCs, but not tenocytes, were capable of differentiating into non-tenocyte lineages of cells suggests that TSCs may play a key role in tendinopathy. While the pathogenesis of tendinopathy is still not clear, previous studies have identified typical histological features including accumulation of lipid cells, glycosaminoglycan accumulation, and tissue calcification, either alone or in combination [17]. Therefore, by virtue of their ability to differentiate into adipocytes, chondrocytes, and osteocytes, TSCs could be responsible for the production of abnormal matrix components (e.g. fatty degeneration, glycosaminoglycan accumulation, and calcifications) seen in tendinopathic tendons. This is an intriguing hypothesis that should be tested in future studies. In addition to TSCs, however, tenocytes may well be involved in the development of tendinopathy through production of inflammatory mediators [18-20] and tissue degradative enzymes (MMPs)[21].

A few comments are in order regarding the stem cell markers identified on TSCs but not on tenocytes in this study. Oct-4 is a transcription factor that is typically expressed in embryonic stem cells during development and is essential for establishing and maintaining undifferentiated pluripotent stem cells [22]. Like previous studies that showed Oct-4 expression in human and mouse BMSCs [23-25], we also found that TSCs expressed Oct-4, encouraging future examination of whether the multi-potency of TSCs demonstrated in this study depends on Oct-4 expression.

In addition to Oct-4 expression, we found that SSEA-4 was consistently expressed in TSCs at low (< 2) and high passages (~12) even after long term culturing. It is known that SSEA is developmentally regulated during early embryogenesis and is widely used as a marker to monitor the differentiation of both mouse and human embryonic stem cells [26,27]. Therefore, SSEA-4 may be used as one of TSC markers. Finally, we found that nucleostemin was highly expressed in rabbit TSCs. Nucleostemin is only expressed in the nucleoli of stem cells and cancer cells, but not in those of committed and terminally differentiated cells [28]. Thus, the high levels of nucleostemin expression in rabbit TSCs indicate that TSCs were an actively proliferating, self-renewing population of cells in our culture conditions. On the other hand, the lack of expression of nucleostemin in tenocytes suggests that tenocytes are terminally differentiated cells without further differentiation potential, as indicated by our data. Finally, as this study shows that TSCs, but not tenocytes, express Oct-4, SSEA-4, and nucleostemin, they may be used as markers to detect these tendon stem cells *in situ*.

A few comments are also necessary regarding cell cultures used in this study. First, TSCs are most likely a mixture of stem cells and progenitor cells, which are heterogeneous in clonogenicity, multi-differentiation potential, and self-renewal. The evidence for this include: 1) colony size of both PTSCs and ATSCs varied greatly (Figure 1); 2) PTSCs and ATSCs did not undergo complete differentiation into adipocytes, chondrocytes, and osteocytes when grown in respective induction media (Figure 2A-H); and 3) not all individual cells were noted to express stem cell markers, including Oct-4 and SSEA-4 (Figure 6). Future studies should look into the properties (e.g. gene expression profiles) of TSCs at the individual cell level rather than at the population level as in this study. Second, when tenocytes were exposed to induction medium for adipogenesis, a small percentage of cells (< 5%) formed lipid drops (Figure 2I, M). These positively-stained cells were likely those remaining TSCs and/or their committed progenitor cells, as it is difficult to isolate pure tenocytes from TSCs using the separation procedures used in this study.

Tendons are commonly considered to contain only tenocytes or tendon fibroblasts [18,20,29]. While it was suggested that there might be a special cell population within tendons that possesses multiple differentiation potential [30], the existence of stem cells in human and mouse tendons was not definitively shown until a recent study [9]. Our finding that rabbit patellar and Achilles tendons contain stem cells is consistent with this study. However, our finding that tenocytes do not have multi-differentiation potential is inconsistent with an earlier study, which showed that so called tendon-derived fibroblasts can differentiate into adipocytes, chondrocytes, and osteocytes [10]. Different tissue culture techniques may account for this discrepancy. Specifically, we used a tissue digestion method to isolate tendon cells directly from tendon tissues. Once tendon cells in culture attached to culture plates, we separated colony-forming cells, which were TSCs, from those cells that spread out, which were considered to be tenocytes. In the study by de Mos et al., tendon explant cultures were used in which cells that migrated out from tendon samples were collected and sub-cultured. Therefore, we suspect that such cultures contained a mixed population of cells including tenocytes and TSCs or their progeny cells. Consequently, those cells that were shown to differentiate into non-tenocytes could actually be TSCs.

While this study shows that TSCs from both patellar and Achilles tendons exhibit similar patterns in directed differentiation and gene expression, they display marked differences in colony formation and cell proliferation rate. In particular, PTSCs formed more and larger colonies (Figure 1A) and proliferated more rapidly than ATSCs (Figure 7). The reasons for these differences are not clear but may reflect inherent differences between the two tendons *in vivo*. The patellar tendon is similar to a ligament as it connects the patella and tibia, and the structure and composition of the patellar and Achilles tendons are different. Our findings of biological differences between patellar and Achilles TSCs support the notion that characteristics of adult stem cells such as TSCs are tissue origin-dependent [31]. Furthermore, the differential proliferative properties of TSCs found in patellar and Achilles tendons may reflect differences in their function and regenerative potential *in vivo*.

## Conclusions

This study showed that TSCs differ from tenocytes in morphology in culture, proliferative potential, and expression of stem cell markers (Oct-4, SSEA-4, and nucleostemin). Moreover, unlike tenocytes, TSCs were shown to possess multi-differentiation potential. Future research should determine whether TSCs can be used for more effective repair or possibly for regeneration of tendinopathic tendons. In addition, considering that tendons are constantly subjected to mechanical loading, future studies should look into the mechanobiology of TSCs and the interactions of TSCs with tenocytes, so that tendon physiology and pathology (e.g. tendinopathy) can be better understood.

## Competing interests

The authors declare that they have no competing interests.

## Authors' contributions

JZ performed experiments, assembled data, and assisted in drafting the manuscript. JHW initiated the study, performed data analysis, and drafted the manuscript. Both authors have read and approved the final manuscript.

## Pre-publication history

The pre-publication history for this paper can be accessed here:

http://www.biomedcentral.com/1471-2474/11/10/prepub
